# Changes in drowning mortality rates and quality of reporting from 2004–2005 to 2014–2015: a comparative study of 61 countries

**DOI:** 10.1186/s12889-019-7749-2

**Published:** 2019-10-28

**Authors:** Ching-Yi Lin, Liang-Yi Wang, Tsung-Hsueh Lu

**Affiliations:** 1 Institute of Medicine, Chung-Shan Medical University, Taichung, Taiwan; 2 NCKU Research Center for Health Data and Department of Public Health, College of Medicine, National Cheng Kung University, No. 1, Dah Hsueh Road, Tainan, 701 Taiwan

**Keywords:** Drowning, Accidents, Death certificate, Mortality, International comparisons

## Abstract

**Background:**

This study assessed international variations in changes in drowning mortality rates and the quality of reporting specific information in death certificates over the past decade.

**Methods:**

Drowning mortality data of 61 countries were extracted from the World Health Organization Mortality Database. We calculated the percentage change (PC) in age-standardized drowning mortality rates and percentage of drowning deaths reported with unspecified codes between 2004 and 2005 and 2014–2015.

**Results:**

Of the 61 countries studied, 50 exhibited a reduction in drowning mortality rates from 2004 to 2005 to 2014–2015. Additionally, five countries—Lithuania, Moldova, Kyrgyzstan, Romania, and El Salvador—with a high mortality rate in 2004–2005 (> 40 deaths per 100,000) showed improvement (PC < − 32%). By contrast, four countries—South Africa, Guyana, Morocco, and Guatemala—exhibited a more than twofold increase in mortality rates. Regarding the quality of reporting, 34 countries exhibited a decrease in the percentage of unspecified codes. Additionally, three countries—Paraguay, Serbia, and Croatia—with moderate and high percentages of unspecified codes (> 40%) exhibited a marked reduction (PC < − 60%), whereas three countries—Malaysia, Belgium, and Nicaragua—exhibited a notable increase.

**Conclusions:**

Large international variations in the extent of changes in drowning mortality rates and the quality of reporting specific information on the death certificate were observed during the study period.

## Background

According to the Global Burden of Disease 2016 Study (GBD 2016), an estimated 302,900 people died from drowning in 2016, and more than 90% of them were from low- and middle-income countries [1]. According to the Global Report on Drowning, the key risk factors for drowning deaths are lack of barriers controlling exposure to water bodies and lack of adequate supervision for infants and young children. Additionally, poor swimming skills and lack of awareness are contributing factors to drowning deaths. In addition, high-risk behaviour, including consuming alcohol while swimming, may lead to drowning among young individuals and adults. Other risk factors are transport on water and water crossings, lack of safe water supply, and floods [2]. The World Health Organization (WHO), therefore, has suggested 10 effective measures to prevent drowning deaths, ranging from community-based solutions such as day care for children and barriers controlling access to water to effective national policies and legislations regarding water safety including setting and enforcing boating, shipping, and ferry regulations [2].

Fortunately, the age-standardized drowning mortality rate declined from 5.8 (deaths per 100,000 people) in 2006 to 4.2 in 2016, representing a − 27% change [1]. However, little is known regarding international variations in the extent of changes in drowning mortality rates. A recent study using the WHO Mortality Database examined the changes in drowning mortality rates across 21 countries meeting the following four criteria: 1) the death registration system covers 70% of the national population; 2) data are available for 10 years or more for the period between 2000 and 2013; 3) at least 100 drowning deaths have been reported for people under 20 years of age for each year; and 4) the International Classification of Disease, Tenth Edition (ICD-10) was used to code deaths for data analysis. The study reported a reduction in drowning mortality rates in 20 of the 21 countries during 2000–2013 (ranging from − 80% to − 13%) [3]. However, this study investigated only decedents under 20 years of age in 21 countries. In 20 among the total 60 countries studied, more than 50% of the drowning deaths were reported among middle-aged and older adults [4]. The circumstances of exposure to different water body types and implementation of prevention measures varied across different age groups and countries. Therefore, we hypothesize that drowning mortality rates vary when considering all age groups rather than considering only the young age group.

The quality of reported mortality data was investigated in the GBD 2016. The quality of morality data was assessed based on the completeness of death registration, fraction of deaths assigned to “garbage” codes, and fraction of deaths assigned to detailed GBD codes [1]. The GBD 2016 did not define “garbage” codes for drowning deaths. Some investigators have suggested the use of the ICD-10 code W74, “unspecified drowning and submersion,” as an indicator of the quality of reporting specific information on the death certificate [5–7]. A previous study indicated that the percentage of unspecified codes exceeded 50% for 32 of the 69 countries studied [7]. A study revealed an improvement in the quality of reporting specific information in some countries during 2000–2013 but a deterioration in the quality in other countries [3].

In this study, we assessed international variations in drowning mortality rates and the percentage of reporting unspecified codes for drowning deaths among all age groups across countries by using mortality data of the past decade. An international comparison of injury-related mortality rates could identify not only the unique health problem of a country but also successful prevention measures [8–13]. In particular, we aimed to identify the countries previously having high drowning mortality rates and the percentage of unspecified codes that showed a prominent improvement in the two indicators during the past decade, which could help discern some success stories. Additionally, we aimed to identify countries that exhibited a deterioration in the two indicators; drowning should be considered a high-priority public health concern in these countries.

## Methods

Drowning mortality data for 2004–2005 and 2014–2015 were extracted from the WHO Mortality Database [14]. To ensure stability in comparing the mortality rates between 2004 and 2005 and 2014–2015, only the countries with absolute drowning deaths ≥50 in the same period were included in this study. For some countries, the latest available data might be available for 2012–2013 or 2013–2014. Many countries with high drowning deaths, such as China, India, Bangladesh, Pakistan, Nigeria, and Vietnam, did not submit mortality data to the WHO; therefore, these countries were not included in this study.

We used ICD-10 codes W65–W74 to identify drowning deaths. The age-standardized death rate (deaths per 100,000 people) of each country was calculated based on the world standard population [15]. The quality of reporting specific information on the death certificate was defined as the percentage of drowning deaths with the ICD-10 code W74, “unspecified drowning and submersion.” The higher the percentage, the poorer the reporting quality.

The percentage change (PC) in the age-standardized mortality rate and unspecified codes between 2004 and 2005 and 2014–2015 were calculated to assess the extent of change. Countries were trichotomized as good, fair, and poor in terms of improvement in the two indicators based on the PC. For countries with a drastic increase and decline in drowning mortality rates between 2004 and 2005 and 2014–2015 and countries with a higher ranking during 2014–2015, we further examined the changes in drowning mortality rates by age group.

Data visualization techniques were used to more efficiently illustrate the changes. We used a bubble-scatter plot to contrast the changes in mortality rates and percentage of unspecified codes between 2004 and 2005 (X axis) and 2014–2015 (Y axis), with the size of the bubble indicating the number of drowning deaths in each country during 2014–2015. Subsequently, we used a slopegraph to illustrate the changes in country rankings (the smaller the rank, the lower the mortality rates and percentage of unspecified codes) from 2004 to 2005 to 2014–2015. A downward slope indicates a decline in rankings. The ranking of some countries declined despite the decrease in mortality rates and percentage of unspecified codes. We used different colours to indicate the absolute change in mortality rates and percentages; blue indicates a decrease and orange indicates an increase. The dashboards can be accessed at: https://public.tableau.com/profile/robert.lu#!/vizhome/Drowningmortality/Story with permission.

## Results

The numbers of all and unspecified drowning deaths, age-standardised mortality rate, and percentage of unspecified codes for each studied country during 2004–2005 and 2014–2015 as well as the PC from 2004 to 2005 to 2014–2015 are presented in Table 1.

**Table 1:** Numbers of all and unspecified (Uspe) drowning deaths, age-standardized mortality rate (deaths per 100,000 people), percentage of unspecified codes (%), and from 2004 to 2005 to 2014–2015 in 61 countries

	2004–2005				2014–15				PC (%)	
Countries	All	Uspe	Rate	%	All	Unspe	Rate	%	Rate	Unspe
Argentina	1130	720	14.3	64	786	458	9.1	58	−36	−9
Australia	390	76	9.7	19	376	56	7.7	15	−21	− 24
Austria	164	24	8.5	15	69	13	2.8	19	− 67	+ 29
Belgium	151	54	6.4	36	152	119	5.5	78	−13	+ 119
Brazil	12,602	5529	33.0	44	10,530	3912	25.1	37	−24	−15
Bulgaria^a^	365	215	22.8	59	271	152	17.5	56	−23	−5
Canada^b^	582	227	9.0	39	601	131	8.0	22	−11	− 44
Chile	1001	874	31.0	87	665	561	18.0	84	−42	−3
Colombia	2134	834	23.9	39	1547	176	16.1	11	−33	−71
Costa Rica	275	179	32.2	65	212	209	22.1	99	−31	+ 51
Croatia	152	64	14.9	42	175	4	15.4	2	+ 3	−95
Cuba	528	6	23.7	1	433	5	18.6	1	−22	+ 2
Czech	368	200	15.8	54	307	94	11.8	31	− 25	−44
Denmark	85	9	6.6	11	59	6	3.7	10	−44	−4
Ecuador	841	469	31.2	56	700	277	21.9	40	−30	−29
Egypt	2762	934	17.5	34	3555	1217	18.5	34	+ 6	+ 1
El Salvador^a^	517	517	43.6	100	372	294	29.6	79	−32	−21
Estonia	144	44	47.8	31	100	8	32.6	8	−32	−74
Finland	287	5	22.2	2	226	0	14.6	0	−34	−100
France	2157	1778	15.0	82	1773	1450	10.5	82	−30	−1
Georgia	90	43	8.9	48	142	80	16.9	56	+ 90	+ 18
Germany	801	311	4.2	39	833	280	4.1	34	−2	−13
Guatemala	197	194	7.8	98	647	647	20.3	100	+ 161	+ 2
Guyana^b^	43	42	28.5	98	196	196	133.4	100	+ 368	+ 2
Hong Kong	80	14	4.9	18	80	4	3.9	5	−19	−71
Hungary	372	60	16.5	16	278	12	12.7	4	−23	−73
Israel	93	91	6.7	98	83	80	4.7	96	−29	−1
Italy	759	527	5.6	69	696	544	4.4	78	−21	+ 13
Japan	11,806	1196	26.0	10	14,992	1697	24.5	11	−6	+ 12
Kyrgyzstan	539	295	53.9	55	422	226	35.7	54	−34	−2
Latvia	435	51	93.0	12	301	6	64.3	2	−31	− 83
Lithuania	697	17	95.6	2	375	32	53.2	9	−44	+ 250
Malaysia	740	290	13.8	39	525	480	8.5	91	−38	+ 133
Mauritius	70	49	28.8	70	86	86	33.1	100	+ 15	+ 43
Mexico	4452	2313	20.4	52	4105	1506	16.1	37	−21	−29
Morocco^a^	167	99	2.8	59	530	392	7.6	74	+ 177	+ 25
Netherlands	189	116	5.4	61	160	71	3.9	44	−28	−28
New Zealand^b^	134	5	16.8	4	106	10	11.8	9	−30	+ 153
Nicaragua	369	144	35.1	39	281	214	23.6	76	−33	+ 95
Norway	164	124	15.8	76	121	100	9.1	83	−42	+ 9
Panama	247	11	36.9	4	217	28	27.4	13	−26	+ 190
Paraguay^a^	205	149	18.0	73	338	79	25.5	23	+ 41	−68
Peru	878	750	16.4	85	662	605	10.7	91	−35	+ 7
Poland	1998	711	23.9	36	1501	46	17.0	3	−29	−91
Portugal^a^	339	312	14.5	92	159	101	6.3	64	− 56	−31
Korea	1820	677	18.9	37	1153	592	9.2	51	−51	+ 38
Moldova	568	352	77.8	62	364	204	47.6	56	−39	−10
Romania	2207	1143	47.9	52	1362	716	30.6	53	−36	+ 2
Serbia	187	110	11.7	59	156	36	9.3	23	−20	−61
Slovakia^a^	277	28	24.4	10	273	26	21.8	10	−10	−6
South Africa	221	206	2.0	93	2990	2983	26.5	100	+ 1195	+ 7
Spain	1066	715	10.7	67	864	408	7.2	47	−33	−30
Sweden	197	95	8.3	48	204	106	7.7	52	−7	+ 8
Switzerland	83	59	5.3	71	106	81	5.4	76	+ 3	+ 7
Taiwan	1136	1035	24.2	91	697	521	12.6	75	− 48	−18
Thailand	8660	8647	68.8	100	6818	6757	49.4	99	−28	−1
U.K.	420	75	3.3	18	535	166	3.8	31	+ 17	+ 74
Uruguay	146	135	21.5	92	153	150	20.9	98	−3	+ 6
U.S.A.	6890	1580	11.8	23	7008	983	10.7	14	−10	− 39
Uzbekistan^a^	2077	1103	36.8	53	1745	750	28.3	43	−23	−19
Venezuela^b^	1193	366	22.4	31	868	306	14.1	35	− 37	+ 15

^a^Latest available data were for 2013–2014, and the corresponding 10-year-old data were for 2003–2004

^b^Latest available data were for 2012–2013, and the corresponding 10-year-old data were for 2002–2003

**Fig. 1:**
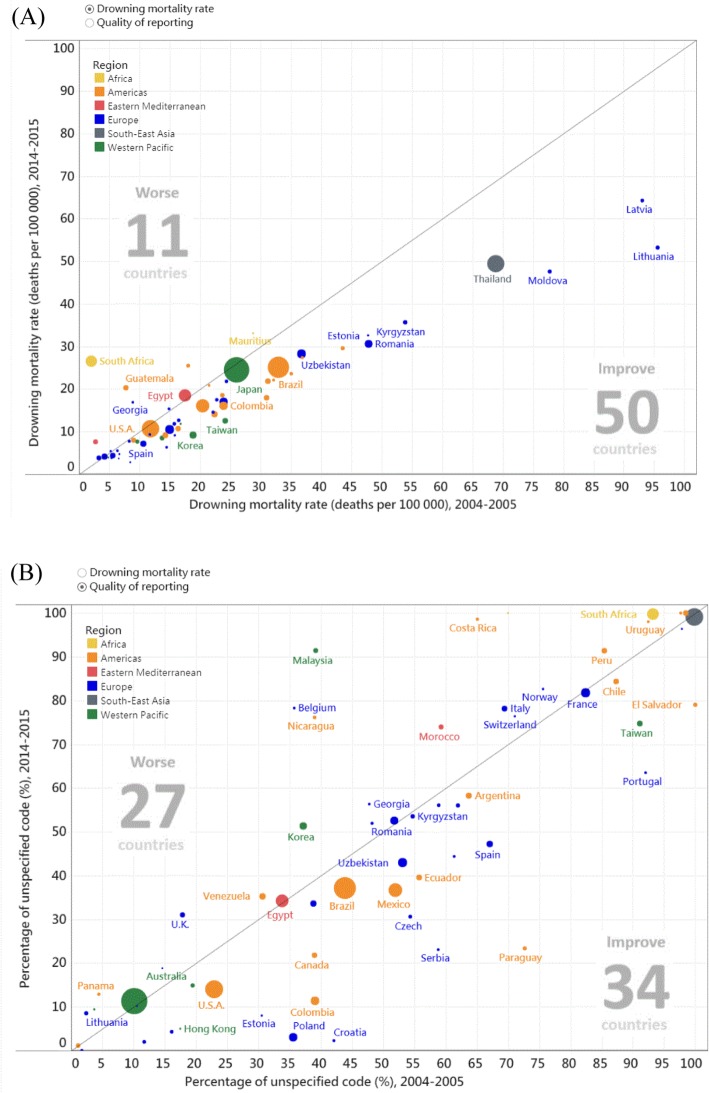
Bubble-scatter plot: **a** drowning mortality rates (deaths per 100,000 people) and (**b**) reporting quality (percentage of drowning deaths with unspecified codes) of the studied countries, 2004–2005 versus 2014–2015 (size of the bubble indicates number of drowning deaths in 2014–2015). https://public.tableau.com/profile/robert.lu#!/vizhome/Drowningmortality/Story with permission

Among the 61 countries studied, 50 exhibited a decline in drowning mortality rates from 2004 to 2005 to 2014–2015, which is indicated by their position below the diagonal line in Fig. 1a. To properly interpret the PC (e.g., some high PCs are attributed to small mortality rates at the 2004–2005 baseline), we classified the countries into high, moderate, and low on the basis of mortality rates (Table 2). Among the countries studied, five countries (Lithuania, Moldova, Kyrgyzstan, Romania, and El Salvador) with high mortality rates during 2004–2005 (> 40 deaths per 100,000) exhibited improvements (PC < − 32%) from 2004 to 2005 to 2014–2015 (Table 2), which can be prominently noted on the right lower side in Fig. 1a, whereas four countries revealed a twofold or more increase in mortality rates, namely South Africa (from 2.0 to 27, + 1195%), Guyana (from 29 to 133, + 368%), Morocco (from 2.8 to 7.6, + 177%), and Guatemala (from 7.8 to 20, + 161%) (Table 2).

**Table 2:** Countries classified according to drowning mortality rate in 2004–2005 and the PC from 2004 to 2005 to 2014–2015

		Improvement of mortality rates based on the PC from 2004 to 05 to 2014–15		
		Good (PC < − 32%)	Fair (PC − 32 to − 20%)	Poor (PC > − 20%)
Drowning mortality rate in 2004–2005, deaths per 100,000 people	High (> 24)	Lithuania (96 to 53, − 44%); Moldova (78 to 48, −39%); Kyrgyzstan (54 to 36, − 34%); Romania (48 to 31, − 36%); El Salvador (44 to 30, − 32%); Nicaragua (35 to 24, − 33%); Chile (31 to 18, − 42%); Taiwan (24 to 13, −48%)	Latvia (93 to 64, − 31%); Thailand (69 to 49, − 28%); Estonia (48 to 33, − 32%); Panama (37 to 27, − 26%); Uzbekistan (37 to 28, − 23%); Brazil (33 to 25, − 24%); Costa Rica (32 to 22, − 31%); Ecuador (31 to 22, − 30%)	Mauritius (29 to 33, + 15%); Guyana (29 to 133, + 368%)*; Japan (26 to 25, − 6%); Slovakia (24 to 22, − 10%)
	Moderate (12–24)	Colombia (24 to 16, − 33%); Venezuela (22 to 14, − 37%); Finland (22 to 15, − 34%); Korea (19 to 9, − 51%); Peru (16 to 11, − 35%); Norway (16 to 9, − 42%); Portugal (14 to 6, − 56%)*; Argentina (14 to 9, − 36%); Malaysia (14 to 9, − 38%)	Poland (24 to 17, − 29%); Cuba (24 to 19, − 22%); Bulgaria (23 to 17, − 23%); Mexico (20 to 16, − 21%); New Zealand (17 to 12, − 30%); Hungary (16 to 13, − 23%); Czech (16 to 12, − 25%); France (15 to 11, − 30%)	Uruguay (22 to 21, − 3%); Paraguay (18 to 25, + 41%); Egypt (18 to 19, + 6%); Croatia (15 to 15, + 3%)
	Low (< 12)	Spain (11 to 7, − 33%); Austria (8 to 3, − 67%); Denmark (7 to 4, − 44%)	Serbia (12 to 9, − 20%); Australia (10 to 8, − 21%); Israel (7 to 5, − 29%); Italy (6 to 4, − 21%); Netherlands (5 to 4, − 28%)	U.S.A. (12 to 11, − 10%); Canada (9 to 8, − 11%); Georgia (9 to 17, + 90%); Sweden (8 to 8, − 7%); Guatemala (8 to 20, + 161%); Belgium (6 to 6, − 13%); Switzerland (5 to 5, + 3%); Hong Kong (5 to 4, − 19%); Germany (4 to 4, − 2%); U.K. (3 to 4, + 17%); Morocco (3 to 8, + 177%)*; South Africa (2 to 27, + 1195%)

**Fig. 2:**
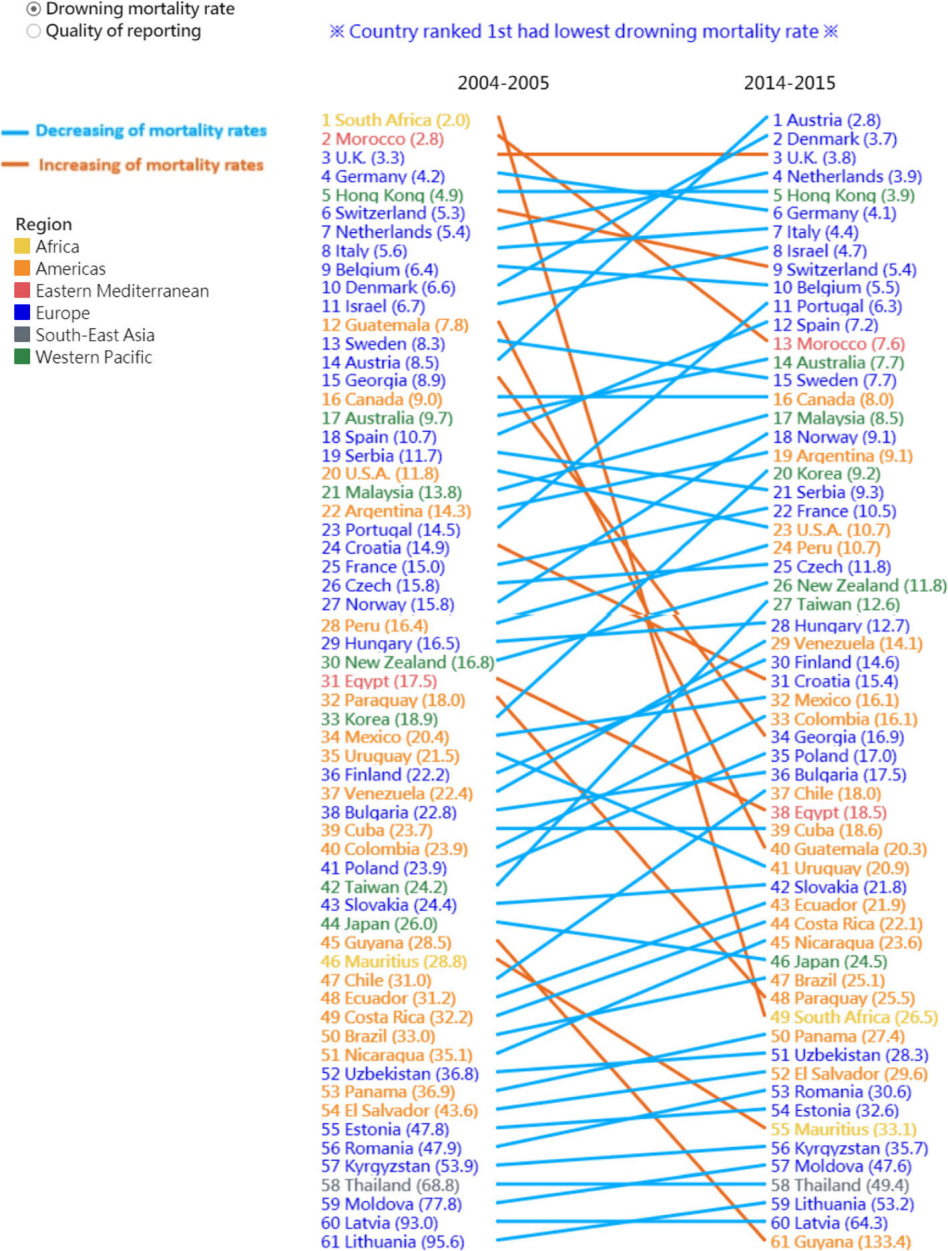
Slopegraph: changes in the ranking of the studied countries with respect to drowning mortality rates (deaths per 100,000 people) from 2004 to 2005 to 2014–2015 (blue indicates a decrease in mortality rates and orange indicates an increase in mortality rates). https://public.tableau.com/profile/robert.lu#!/vizhome/Drowningmortality/Story with permission

Despite 50 countries exhibiting a decline in drowning mortality rates, only 38 countries were ranked higher during 2014–2015 than during 2004–2005 (Fig. 2). We also found that among the 10 top-ranking (first to tenth) countries in 2014–2015, nine were European countries. Notably, although some countries exhibited a decline in ranking (downward slope) (i.e., Germany [fourth to sixth], Belgium [ninth to tenth], Sweden [thirteenth to fifteenth], Serbia [nineteenth to twenty-first], Uruguay [thirty-fifth to forty-first], and Japan [forty-fourth to forty-sixth]), their mortality rates showed a decreasing trend (blue line).

Regarding the reporting quality, 34 countries exhibited a reduction in the percentage of unspecified codes (Fig. 1b). The countries were classified on the basis of their percentage of unspecified codes during 2004–2005 and the PC from 2004 to 2005 to 2014–2015 (Table 3). Three countries (i.e., Paraguay [from 73 to 23, − 68%], Serbia [from 59 to 23, − 61%], and Croatia [from 42 to 2, − 95%)] with moderate and high percentages of unspecified codes (> 40%) during 2004–2005 exhibited a notable reduction (PC < − 60%), which can be prominently noted on the right lower side in Fig. 1b. By contrast, countries with a notable increase in the percentage were Malaysia (from 39 to 91, + 133%), Belgium (from 36 to 78, + 119%), and Nicaragua (from 39 to 76, + 95%) (Table 3).

**Table 3:** Countries classified according to the percentage of unspecified codes in 2004–2005 and the PC from 2004 to 2005 to 2014–2015

		Improvement of quality of reporting based on the PC from 2004 to 05 to 2014–15		
		Good (PC < − 20%)	Fair (PC − 20 to + 7%)	Poor (PC > + 7%)
Percent of unspecified code in 2004–2005,%	High (> 62)	El Salvador (100 to 79, − 21%); Portugal (92 to 64, − 31%); Paraguay (73 to 23, − 68%); Spain (67 to 47, − 30%)	Thailand (100 to 99, − 1%); Guatemala (98 to 100, + 2%); Israel (98 to 96, − 1%); Guyana (98 to 100, + 2%); Uruguay (92 to 98, + 6%); Taiwan (91 to 75, − 18%); Chile (87 to 84, − 3%); Peru (85 to 91, + 7%); France (82 to 82, − 1%); Argentina (64 to 58, − 9%)	South Africa (93 to 100, + 7%); Norway (76 to 83, + 9%); Switzerland (71 to 76, + 7%); Mauritius (70 to 100, + 43%); Italy (69 to 78, + 13%); Costa Rica (65 to 99, + 51%)
	Moderate (37–62)	Netherlands (61 to 44, − 28%); Serbia (59 to 23, − 61%); Ecuador (56 to 40, − 29%); Czech (54 to 31, − 44%); Mexico (52 to 37, − 29%); Croatia (42 to 2, − 95%); Colombia (39 to 11, − 71%); Canada (39 to 22, − 44%)	Moldova (62 to 56, − 10%); Bulgaria (59 to 56, − 5%); Kyrgyzstan (55 to 54, − 2%); Uzbekistan (53 to 43, − 19%); Romania (52 to 53, + 2%); Brazil (44 to 37, − 15%); Germany (39 to 34, − 13%)	Morocco (59 to 74, + 25%); Sweden (48 to 52, + 8%); Georgia (48 to 56, + 18%); Malaysia (39 to 91, + 133%)*; Nicaragua (39 to 76, + 95%); Korea (37 to 51, + 38%)
	Low (< 37)	Poland (36 to 3, −91%); Estonia (31 to 8, − 74%); U.S.A. (23 to 14, − 39%); Australia (19 to 15, − 24%); Hong Kong (18 to 5, − 71%); Hungary (16 to 4, − 73%); Latvia (12 to 2, − 83%); Finland (2 to 0, − 100%)*	Egypt (34 to 34, + 1%); Denmark (11 to 10, − 4%); Slovakia (10 to 10, − 6%); Cuba (1 to 1, + 2%)	Belgium (36 to 78, + 119%); Venezuela (31 to 35, + 15%); U.K. (18 to 31, + 74%); Austria (15 to 19, + 29%); Japan (10 to 11, + 12%); Panama (4 to 13, + 190%); New Zealand (4 to 9, + 153%)*; Lithuania (2 to 9, + 250%)*

**Fig. 3:**
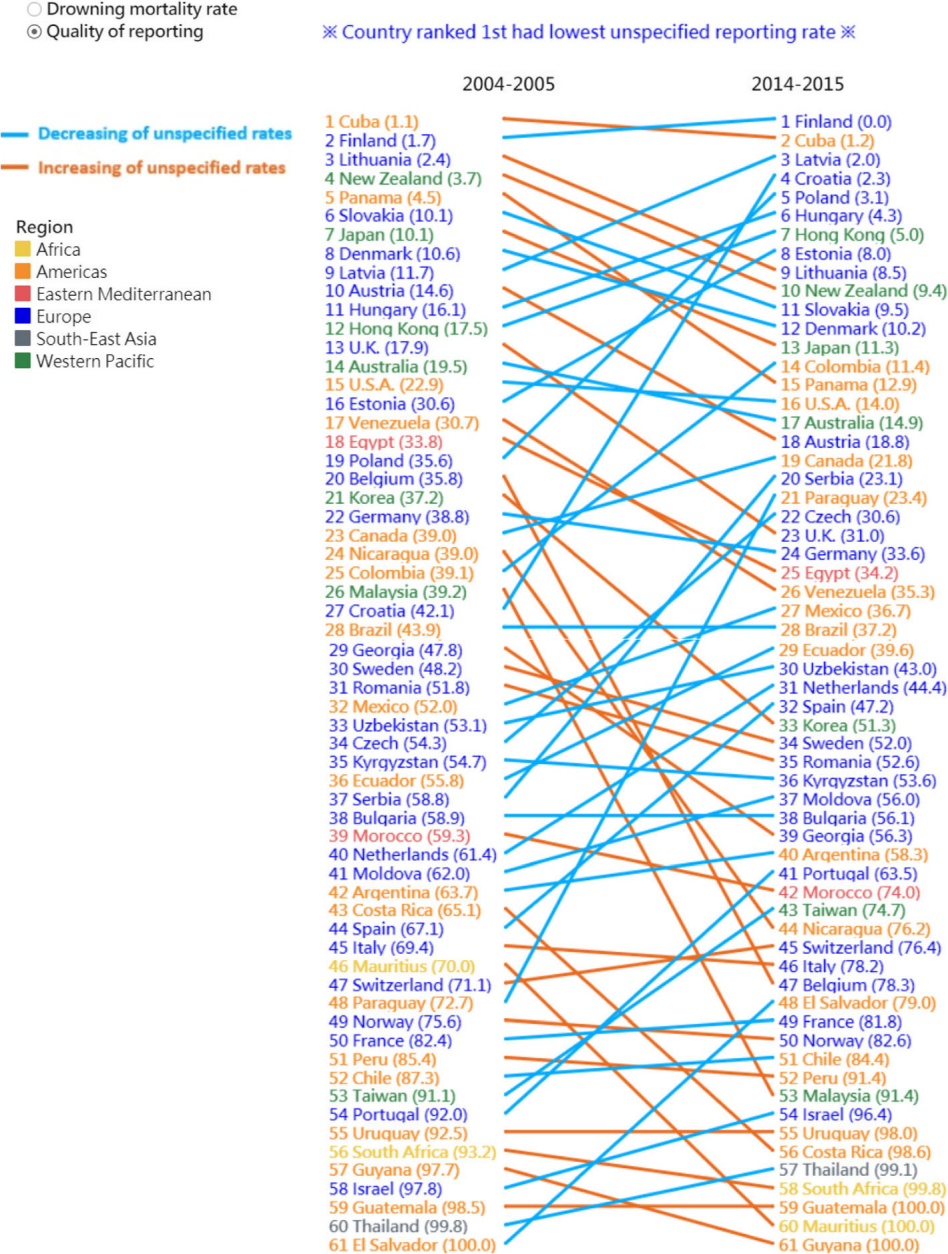
Slopegraph: changes in the ranking of the studied countries in terms of the reporting quality (percentage of drowning deaths with unspecified codes) from 2004 to 2005 to 2014–2015 (blue indicates a decrease in the percentage and orange indicates an increase in the percentage). https://public.tableau.com/profile/robert.lu#!/vizhome/Drowningmortality/Story with permission

Despite 34 countries exhibiting a reduction in the percentage of unspecified codes, only 28 countries exhibited higher rankings during 2014–2015 than during 2004–2005 (Fig. 3). Similarly, during 2014–2015, of the 10 top-ranking (first to tenth) countries, seven were European countries. Notably, although some countries exhibited a decline in rankings (downward slope), namely, Slovakia (from sixth to eleventh), Denmark (from eighth to twelfth), Australia (from fourteenth to seventeenth), United States (from fifteenth to sixteenth), Germany (from twenty-second to eleventh), and Kyrgyzstan (from thirty-fifth to thirty-sixth), the percentage of unspecified codes showed a decreasing trend (blue line).

For countries reporting a drastic increase in drowning mortality rates during the study period (South Africa, Guyana, Morocco, and Guatemala), we further analysed the mortality changes by age group (Table 4). We found that the mortality changes showed a drastic increase across all the age groups. For the three countries (Austria, Portugal, and Korea) showing the largest decline in drowning mortality rates, we found that the extent of the decline was similar in each age group. A total of four European countries (Austria, Demark, the United Kingdom, and the Netherlands) with the highest ranking (lowest mortality rates) in 2014–2015 showed the largest decline for children aged ≤14 years; however, the magnitude of the decline was less prominent for middle-aged and older adults and even increased for people aged 15 years and older in the United Kingdom.

**Table 4:** Changes in drowning mortality rates by age group in selected countries

Countries	Age, years	2004–2005	2014–2015	% change
Countries with largest increase in mortality rates				
South Africa	All ages	2.0	26.5	+ 1195%
	<=14	5.2	39.0	+ 644%
	15–24	0.8	22.6	+ 2740%
	25–44	0.7	20.2	+ 2605%
	45–64	0.9	23.3	+ 2580%
	> = 65	1.9	25.2	+ 1215%
Guyana	All ages	28.5	133.4	+ 368%
	<=14	22.0	55.3	+ 152%
	15–24	27.3	139.9	+ 413%
	25–44	38.4	167.3	+ 336%
	45–64	15.3	202.0	+ 1216%
	> = 65	48.2	83.3	+ 73%
Morocco	All ages	2.8	7.6	+ 177%
	<=14	2.5	7.1	+ 180%
	15–24	4.4	14.0	+ 220%
	25–44	1.8	7.6	+ 316%
	45–64	0.8	3.9	+ 374%
	> = 65	8.0	5.2	+ − 36%
Guatemala	All ages	7.8	20.3	+ 161%
	<=14	4.8	14.0	+ 189%
	15–24	11.5	25.8	+ 124%
	25–44	7.9	22.1	+ 180%
	45–64	9.6	19.6	+ 103%
	> = 65	4.5	24.3	+ 438%
Countries with largest decline in mortality rates				
Austria	All ages	8.5	2.8	−67%
	<=14	4.5	0.8	−82%
	15–24	7.4	4.0	−47%
	25–44	5.8	1.1	−81%
	45–64	14.3	3.5	−76%
	> = 65	18.7	11.7	−38%
Portugal	All ages	14.5	6.3	−56%
	<=14	9.2	2.0	−79%
	15–24	14.4	8.1	−44%
	25–44	13.4	5.7	−57%
	45–64	17.9	9.3	−48%
	> = 65	27.2	11.6	−57%
Korea	All ages	18.9	9.2	−51%
	<=14	18.0	4.4	−75%
	15–24	16.4	5.8	−65%
	25–44	13.4	5.9	−56%
	45–64	20.0	13.0	−35%
	> = 65	43.3	34.2	−21%
Countries with highest ranking (lowest mortality rates) in 2014–2015				
Denmark	All ages	6.6	3.7	−44%
	<=14	3.0	0.5	−82%
	15–24	6.7	2.1	−69%
	25–44	3.2	2.5	−24%
	45–64	12.9	8.0	−38%
	> = 65	14.7	11.4	−23%
U.K.	All ages	3.3	3.8	+ 17%
	<=14	2.4	1.8	−24%
	15–24	3.5	4.9	+ 40%
	25–44	3.1	4.0	+ 29%
	45–64	4.0	4.9	+ 23%
	> = 65	4.6	4.9	+ 6%
Netherlands	All ages	5.4	3.9	−28%
	<=14	7.2	3.5	−51%
	15–24	2.6	2.2	−15%
	25–44	3.0	2.7	−11%
	45–64	6.8	5.0	−27%
	> = 65	10.7	10.3	−4%

## Discussion

The findings of this study indicate that most of the studied countries (50/61) showed a decline in drowning mortality rates during the past decade. However, only half of the studied countries (34/61) exhibited an improvement in the quality of reporting specific information on the death certificate. Some countries demonstrated a larger PC in the two indicators, which could provide insights for preventing drowning deaths.

One possible explanation for the decline in drowning mortality rates in many countries is the proposal and implementation of several evidence-based drowning prevention interventions during the past decade [2, 16–19]. However, Towner et al. indicated that countries with the widest range of injury prevention legislations and strongest enforcements have not made relative progress in reducing child injury deaths since the 1970s [11]. In a historical study, Staines and Ozanne–Smith highlighted other key factors of the reduction in drowning mortality rates (i.e., infrastructural development [e.g., the development of a piped water supply system eliminated the need for reliance on hazardous water supplies or storage systems], urbanization, and mandatory schooling [thereby reducing the amount of time that children were unsupervised and in hazardous environments]) [20].

European countries showed high ranking in both the indicators, for example, nine of the ten highest ranked (lowest mortality rates) countries during 2014–2015 were European countries (Fig. 2). Most of the decline in drowning mortality rates from 2004 to 2005 to 2014–2015 in these countries were attributed to the reductions in child drowning mortality rates and possibly due to the Tools to Address Childhood Trauma, Injury and Children’s Safety funding and partnership coordinated by the European Child Safety Alliance [21]. On July 14, 2008, the European Child Safety Alliance, EuroSafe, launched a new resource titled “Protecting children and youth in water recreation: safety guidelines for service providers” [22]. This water safety resource is particularly optimized for people working in the water recreation and tourism sectors to assist them in offering safe water-related activities and services for children and families throughout Europe.

We found that four countries (South Africa, Guyana, Morocco, and Guatemala) exhibited the highest increases in drowning mortality rates from 2004 to 2005 to 2014–2015, in which an increase occurred in each age group. According to the report of The Global Facility for Disaster Reduction and Recovery, we found tsunami and flood were the possible causes of the increase in the drowning mortality rates in these four countries [23].

Regarding the improvement in the reporting quality of Paraguay, Serbia, and Croatia, we are expecting that some studies may be performed to understand if there were any interventions or efforts implemented during the past decade. The coding for drowning deaths as unspecified may result from various factors, such as lack of specific information on the circumstances that led to the drowning, inadequate collection of primary data owing to insufficient police and medico-legal investigations, and incompleteness or errors during the death certification and coding processes [7]. Information on the concerned water body is paramount in designing relevant prevention programs. For example, in Japan, the main water body for drowning deaths among older adults is the bathtub [24], for which the prevention measures would be quite different from measures required for preventing children from drowning in swimming pools.

Several limitations should be considered while interpreting the findings of this study. First, some countries with high drowning death rates, such as China, India, Bangladesh, Pakistan, Nigeria, and Vietnam, were not included in this study because of the unavailability of recent data or details in the WHO Mortality Database. Second, only two time points (2004–2005 and 2014–2015) were used to examine the changes, and some countries with a relatively small number of drowning deaths might exhibit large fluctuations in mortality rates. To avoid the improper interpretation of the changes, we further analysed the yearly mortality rates to examine whether the pattern of the mortality trend was linear or nonlinear. Third, we did not have detailed contextual information on drowning mortality rates and the reporting quality in each studied country, which impeded a reliable explanation of the changes.

## Conclusions

The study findings indicate large international variations in the extent of changes in drowning mortality rates and the quality of reporting specific information on the death certificate during the past decade. Additional studies are required to thoroughly understand the reasons for the drastic improvement or deterioration in both the indicators.

## Data Availability

The WHO Mortality Database in which this study used can be freely accessed at https://www.who.int/healthinfo/statistics/mortality_rawdata/en/ We declare that the calculation and interpretation of the mortality rates derived from WHO Mortality Database were those of the authors not the WHO.

## Abbreviations

GBD: Global burden of disease
ICD-10: International Classification of Diseases Tenth Revision
WHO: World Health Organization
